# Impact of magnetic fields on small field dosimetry for parallel MR‐linacs

**DOI:** 10.1002/mp.18052

**Published:** 2025-08-13

**Authors:** Ahtesham Ullah Khan

**Affiliations:** ^1^ Department of Medical Physics School of Medicine and Public Health University of Wisconsin‐Madison Madison Wisconsin USA

**Keywords:** magnetic fields, Monte Carlo, MR‐guided RT, MR‐linacs, small field dosimetry

## Abstract

**Background:**

The use of MR‐guided radiation therapy (MRgRT) is increasing with the introduction of commercially‐available parallel MR‐linac systems. Treatment fields used by these machines can be very small and highly modulated. Literature on the small field dosimetry of parallel MR‐linacs and the impact of the magnetic field on dose perturbations is scarce.

**Purpose:**

To investigate the impact of magnetic fields on small field dosimetry of parallel MR‐linacs using Monte Carlo (MC) methods.

**Methods:**

A TOPAS MC model of a 6 MV FFF beam was developed and validated against a commercial 0.5 T parallel MR‐linac. The impact of varying magnetic field of up to 3.0 T on water phantom dosimetry such as percent depth dose (PDD), beam profiles, and output factors (OFs) was studied. A lung phantom with embedded spherical tumors of diameters 1–3 cm was employed to investigate the impact of parallel magnetic fields on lung lesions using an arc delivery with a 4^ο^ gantry spacing providing a surrogate for rotational delivery techniques. Dose distributions were compared with the 0.0 T scenario.

**Results:**

Negligible differences were noted in the PDD, except for the build‐up region, between the investigated parallel magnetic fields. Besides the tail region of the 1.5 and 3.0 T profiles, minimal differences were observed in the lateral beam profiles. Similarly, parallel magnetic fields of up to 1 T had a negligible impact on small field OFs. Compared to the 0 T magnetic field, the OF for field sizes of <2 × 2 cm^2^ was found to increase significantly, by up to 9%, for the 1.5 and 3.0 T fields. The increase in magnetic field strength led to a more uniform dose distribution across the tumor inside the lung phantom with a slight reduction in penumbra due to the electron focusing effect. Normalized to the 0 T lung phantom dose distribution, differences ranging from 3% to 8% were found when the magnetic field strength was varied from 0.5 to 3.0 T. These differences were proportional to the field strength and were more significant for the smaller tumor diameters employing smaller fields.

**Conclusions:**

The impact of parallel magnetic fields of up to 1.0 T strength is minimal for in‐water small field dosimetry. However, the increase in OF, compared to the 0 T case, was found to be significant for the 3.0 T magnetic field. For all field strengths compared to 0 T, significant dose differences were found around the periphery of the tumors inside a lung phantom that must be accounted for during treatment planning and dosimetry.

## 1 INTRODUCTION

Prevalence of magnetic resonance‐guided radiation therapy (MRgRT) has been increasing with the emergence of hypo fractionated treatments and adaptive radiation therapy (ART).^1^ Incorporating a linear accelerator (linac) with an MRI scanner is a monumental engineering challenge with various different approaches taken by multiple groups.^2^, ^3^, ^4^, ^5^ Orthogonal MR‐linacs, such as the ViewRay Systems MRIdian and the Elekta Unity systems, incorporate a uniform external magnetic field that is orthogonal to the radiation beam. The electron return effect (ERE), caused by the Lorentz force influencing the trajectory of the secondary charged particles, skews the dose distribution in the presence of the magnetic field especially at tissue‐lung and tissue‐air interfaces.^6^ Therefore, these effects must be taken into account during treatment planning and dose calculation. Conversely, parallel MR‐linacs, such as Aurora‐RT, orient the radiation beam parallel to the magnetic field eliminating ERE whilst increasing skin dose due to the focusing of contaminant electrons along the direction of the radiation beam.^7^ Both MR‐linac configurations have unique dosimetric challenges that must be considered. This work focuses on the parallel MR‐linac configuration.

Treatment of small thoracic and abdominal lesions using intensity‐modulated radiation therapy (IMRT) is commonplace in MRgRT where small fields are regularly used in treatment planning. Small fields are affected by the source occlusion effect and the lateral charged particle equilibrium (LCPE) conditions.^8^ The presence of a magnetic field further distorts the inequilibrium conditions encountered in small field dosimetry.^9^ Suitable MR‐compatible radiation detectors along with small field correction factors can alleviate some of these challenges. The LCPE, for a given field size, is dependent on the energy of the photon beam as well as the strength and direction of the magnetic field. The presence of a parallel magnetic field focuses the secondary charged particles and diminishes the LCPE conditions encountered in small field dosimetry. Such an effect is more dominant for lesions surrounded by low density media such as lung.^10^

Literature pertaining to small field dosimetry for parallel MR‐linacs is scarce and the impact of the magnetic field on inequilibrium conditions remains to be investigated. Studying the impact of varying magnetic field strengths on small field dosimetry can also assist in the design of future parallel MR‐linacs. Monte Carlo (MC) modeling of a parallel MR‐linac can provide valuable insights related to the influence of an external magnetic field on small field dosimetry conditions. Therefore, the aim of this work was to utilize MC methods to investigate the impact of a parallel magnetic field, ranging from 0T to 3T, on dosimetric data such as percent depth dose (PDD), lateral profiles, and output factors (OFs). Additionally, a lung phantom with three different lesion sizes was employed to study the effect of parallel magnetic fields on the 3D dose distributions created using the IMRT arc technique (Table 1).

**TABLE 1:** Simulation parameters used in Monte Carlo simulations in accordance with AAPM TG‐268.

Item name	Description	References
Code version	TOPAS v3.7 with GEANT4 extensions	11, 12
Validation	Comparison with percent depth dose (PDD) and lateral beam profiles	7
Timing	∼18 h per job	
Source description	Electron beam incident on tungsten target	
Transport parameters	Previously reported in great detail	13, 14
Variance reduction	Directional bremsstrahlung splitting with a 1000 splitting number	
Scored quantity	Absorbed dose to medium	
Statistical uncertainty	<0.50%	
Postprocessing	None	

## 2 METHODS

Throughout this work, all Monte Carlo (MC) simulations were run in sequential mode with 2000 concurrent independent simulation jobs (2000 cores) on the University of Wisconsin‐Madison Center of High Throughput Computing (CHTC) cluster.

### 2.1 Monte Carlo model creation and validation

A MC model of a parallel MR‐linac, mimicking the Aurora‐RT system, was created in TOPAS MC code.^11^, ^15^ Since proprietary information related to the geometrical components of the Aurora‐RT MR‐linac was not available, the MR‐linac model was created by approximating the dimensions and materials of the photon target, collimating jaws, and magnetic field region. The model geometry was tuned based on existing experimental data. The TOPAS code was extended with a custom GEANT4 electromagnetic physics list based on a previous study by Simiele and DeWerd.^14^ Using a Fano cavity test, the electromagnetic physics parameters were optimized to yield the highest accuracy of the condensed history transport algorithm in magnetic fields. The physics parameters used in this study have been described in great details in our previous works.^13^, ^16^ The production threshold for both photon and charged particles were set to 0.5 mm throughout this work. Directional bremsstrahlung splitting (DBS) was utilized as a variance reduction technique with a splitting number of 1000 and a radius larger than the field size.

The MR‐linac model comprised of an electron beam impinging on a tungsten photon target, jaws, and a magnetic field region housing the virtual phantoms. The jaws were modeled to be divergent with the radiation beam at all field sizes and the magnetic field was simulated to be constant throughout the bore volume. All field sizes were defined at the source‐to‐axial distance (SAD) of 120 cm. To be consistent with the MR‐linac design, all contaminant electrons proximal to 60 cm distance from the target were removed from the simulations allowing for similar surface dose between the simulated dose and experimental dose.^17^ The electron beam was simulated as a circular beam with a Gaussian positional and energy distributions. The tunable model parameters were the electron beam average energy and the full width at half maximum (FWHM) of the Gaussian spatial and energy distribution. During the validation phase, a virtual 30×30×30 cm^3^ water phantom was created with 2×2×2 mm^3^ voxel sizes. Experimental PDD and lateral profile data, measured using a PTW semiflex 3D ion chamber, for a 20×20 cm^2^ field were extracted from the work of Oliver et al. and the model parameters were iteratively tuned to match the simulation data with the experimental data.^7^ The model tuning methodology described in our previous work was followed in this work.^13^ The average energy of the Gaussian distribution was tuned first by maximizing the agreement between the simulated and experimental PDD with changes in average energy from 5.5 to 6.5 MeV in 0.2 MeV increments. The jaw opening was adjusted to match the experimental FWHM of the 20×20 cm^2^ field profiles. The FWHM of the Gaussian spatial distribution was varied from 0.5 to 3 mm in 0.5 mm increments to match the simulated profiles with the experimental profiles. The FWHM of the Gaussian energy distribution was varied from 0 to 3 MeV to maximize the model agreement for PDD and profiles. The beam quality metric tissue‐phantom ratio (TPR) of 20 cm depth to 10 cm depth for a reference 10×10 cm^2^ field size was also simulated and compared with the experimental value.^18^ These data were acquired with a 0.5 T magnetic field strength.

### 2.2 Water phantom dosimetry

Using a voxelized 30 × 30 × 30 cm^3^ water phantom, PDD and profiles were simulated with a source‐to‐surface distance (SSD) of 110 cm. Voxel size was set to 2 × 2 × 2 mm^3^ except for the smallest 0.5 × 0.5 cm^2^ field size, where the voxel size was reduced to 0.5 × 0.5 × 0.5 mm^3^ to minimize volume‐averaging effects. Lateral beam profiles were scored at 10 cm depth along with PDD for field sizes of 0.5 × 0.5 cm^2^, 1 × 1 cm^2^, 2 × 2 cm^2^, 3 × 3 cm^2^, and 10 × 10 cm^2^. Output factors (OFs) were calculated, at 5 cm depth and as a function of depth, using the following relationship:

(1) $$OF\left(d,B\right)=\frac{D\left(FS\right)}{D\left(FS_{ref}\right)}$$

where d is depth, D is absorbed dose to water, B is the magnetic field strength, FS is field size, and $FS_{ref}$ is the reference field size of 10 × 10 cm^2^. The FS was defined to be the FWHM of the beam profiles and the geometric mean of the profiles’ FWHM across both direction was calculated to be $S_{clin}$.^19^, ^20^ All simulation data were acquired for magnetic field strengths of 0.0, 0.5, 1.0, 1.5, and 3.0 T. These data were compared to study the impact of magnetic field strength on small field dosimetry.

### 2.3 Lung phantom comparison

**FIGURE 1:**
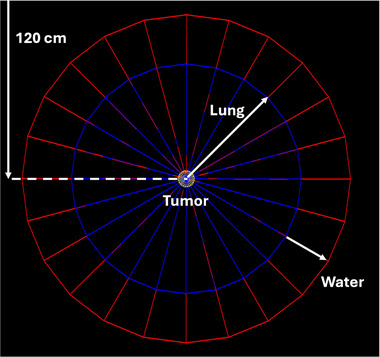
Visual schematic of the virtual lung phantom used in this study.

A virtual cylindrical lung phantom, shown in Figure 1, was modeled with a spherical tumor of varying diameter in the center, surrounded by a lung cylinder of 7 cm radius and a water cylindrical shell of 3 cm thickness. The height of the cylinders was set to 10 cm to provide adequate lateral scatter conditions. The center of the phantom coincided with the isocenter with lung material composition and density extracted from report ICRU‐44.^21^ The density of the lung material was 0.26 g/cc. Spherical tumors of 1, 2, and 3 cm diameters were modeled with square field sizes of 2 × 2 cm^2^, 3 × 3 cm^2^, and 4 × 4 cm^2^, respectively, to provide a 0.5 cm margin around the target volume. The voxel size of 0.5 × 0.5 × 0.5 mm^3^ was employed for the 1 cm tumor sphere and 2 × 2 × 2 mm^3^ was employed for the 2 and 3 cm tumor spheres to ensure minimal volume‐averaging and maximal computational efficiency. These lesions are representative of clinical lung tumors where small fields may be used extensively. IMRT‐type delivery was mimicked by simulating an arc with equally weighted segments and gantry spacing of 4^ο^. 3D dose distributions were acquired for magnetic field strengths of 0.0, 0.5, 1.0, 1.5, and 3.0 T.

## 3 RESULTS

**FIGURE 2:**
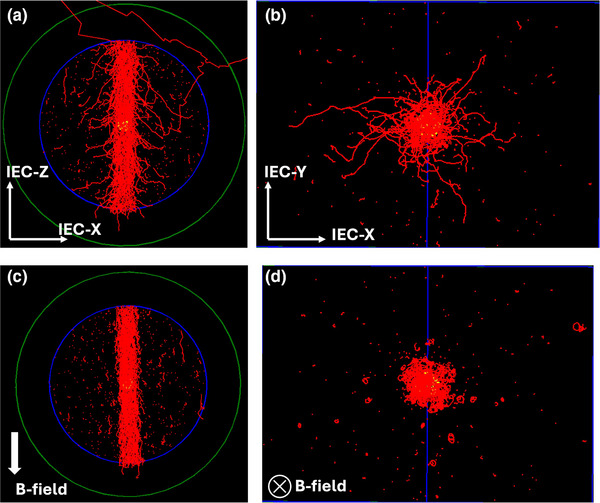
Electron tracks, visualized in TOPAS Monte Carlo code, inside the magnetic field region for 0.0 T (a and b) and 3.0 T (c and d). The focusing effect of the parallel magnetic field and the reduction in lateral electron fluence can be appreciated. The same number of histories were utilized for all subfigures.

Figure 2 displays the electron tracks for 0.0 and 3.0 T magnetic fields. The reduction in lateral charged particle fluence can be visualized, that creates a focusing effect diminishing the lateral inequilibrium of the charged particle fluence.

### 3.1 Model validation

**FIGURE 3:**
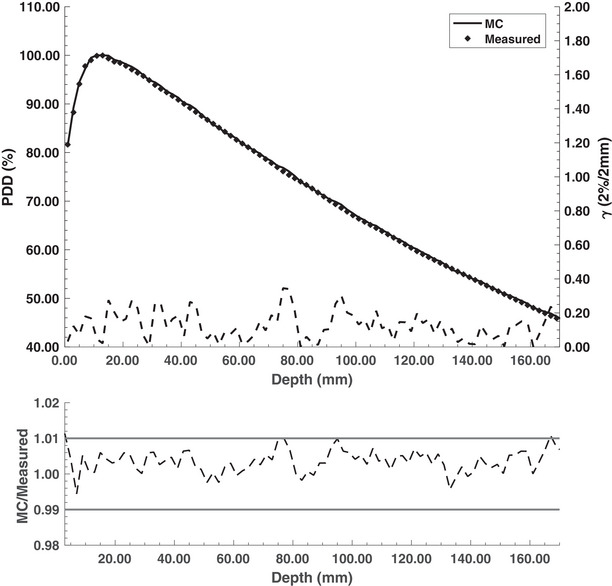
Experimental and Monte Carlo (MC)‐calculated percent depth dose (PDD) curves for the 0.5 T Aurora‐RT MR‐linac with a field size of 20 × 20 cm^2^. The gamma pass rate was 100% with a 2%/2 mm criterion.

**FIGURE 4:**
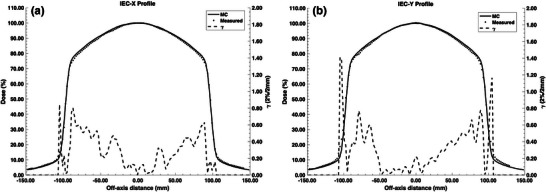
IEC‐X (a) and IEC‐Y (b) profile comparison for the 0.5 T Aurora‐RT MR‐linac between Monte Carlo (MC) and experimental data for a 20 × 20 cm^2^ field size. The gamma pass rate was 100% and 97% for the IEC‐X and IEC‐Y directions, respectively, with a 2%/2 mm criterion.

A 6 MeV mean electron energy with a 1σ energy spread of 0.9 MeV was found to be the optimal model parameters with a 1σ spot size of 1.27 mm. With these parameters, the MC‐calculated PDD comparison with experimental results for a 20 × 20 cm^2^ field size is shown in Figure 3. Excellent agreement, to within 1%, was found between the developed model and the measured results. The $TPR_{20,10}$ for the 10 × 10 cm^2^ reference field size was calculated to be 0.640 and is within 1.3% with the previously published experimental value of 0.632.^18^ As shown in Figure 4, good agreement was also found between the MC‐calculated and experimental beam profiles for a 20 × 20 cm^2^ field size.

### 3.2 Water phantom dosimetry

**FIGURE 5:**
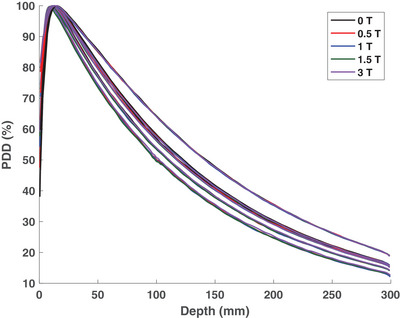
Percent depth dose (PDD) curves for the 0.5 × 0.5, 1 × 1, 2 × 2, 3 × 3, and 10 × 10 cm^2^ field sizes for magnetic field strengths ranging from 0 to 3 T.

The impact of the parallel magnetic field, ranging from 0 to 3 T, on PDD for various field sizes is displayed in Figure 5. Distal to depth of maximum dose, $d_{max}$, differences of <1% were found between the PDD curves for the investigated magnetic field strengths. The build‐up dose for each field size was found to be larger for the higher magnetic field strengths due to the increased focusing effect on the contaminant electrons. This effect also led to a proximal shift in $d_{max}$ of 2 mm for all field sizes except for the 0.5 × 0.5 cm^2^ field size, where a shift of 1 mm was observed. However, minimal differences were noted in the electron contaminant‐free region.

**FIGURE 6:**
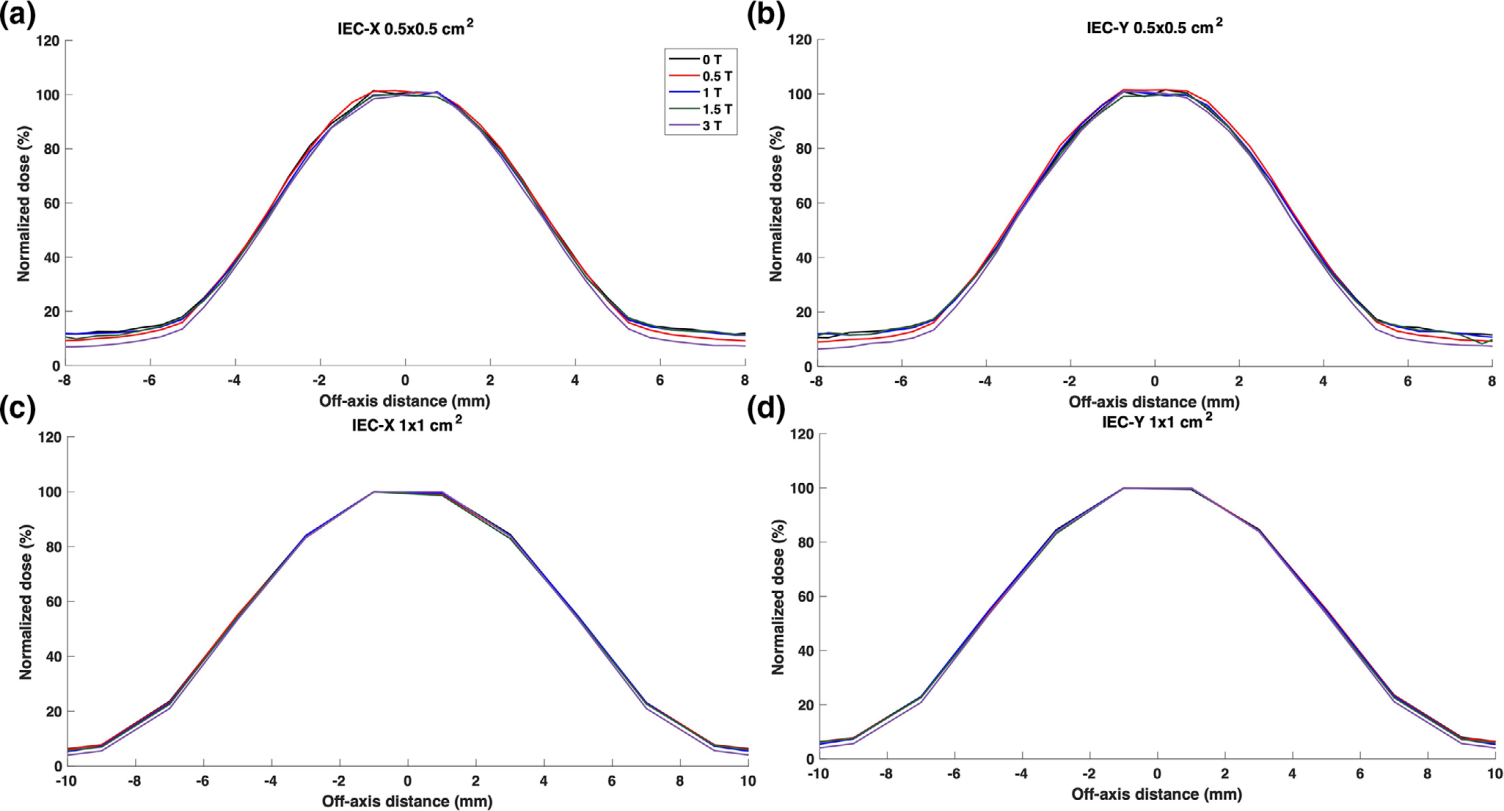
Lateral beam profiles for the 0.5 × 0.5 cm^2^ (a and b) and 1 × 1 cm^2^ (c and d) field sizes along both IEC‐X (a and c) and IEC‐Y (b and d) directions. Profiles are included for parallel magnetic field strengths ranging from 0 to 3 T. The same legend applies to all subfigures.

**FIGURE 7:**
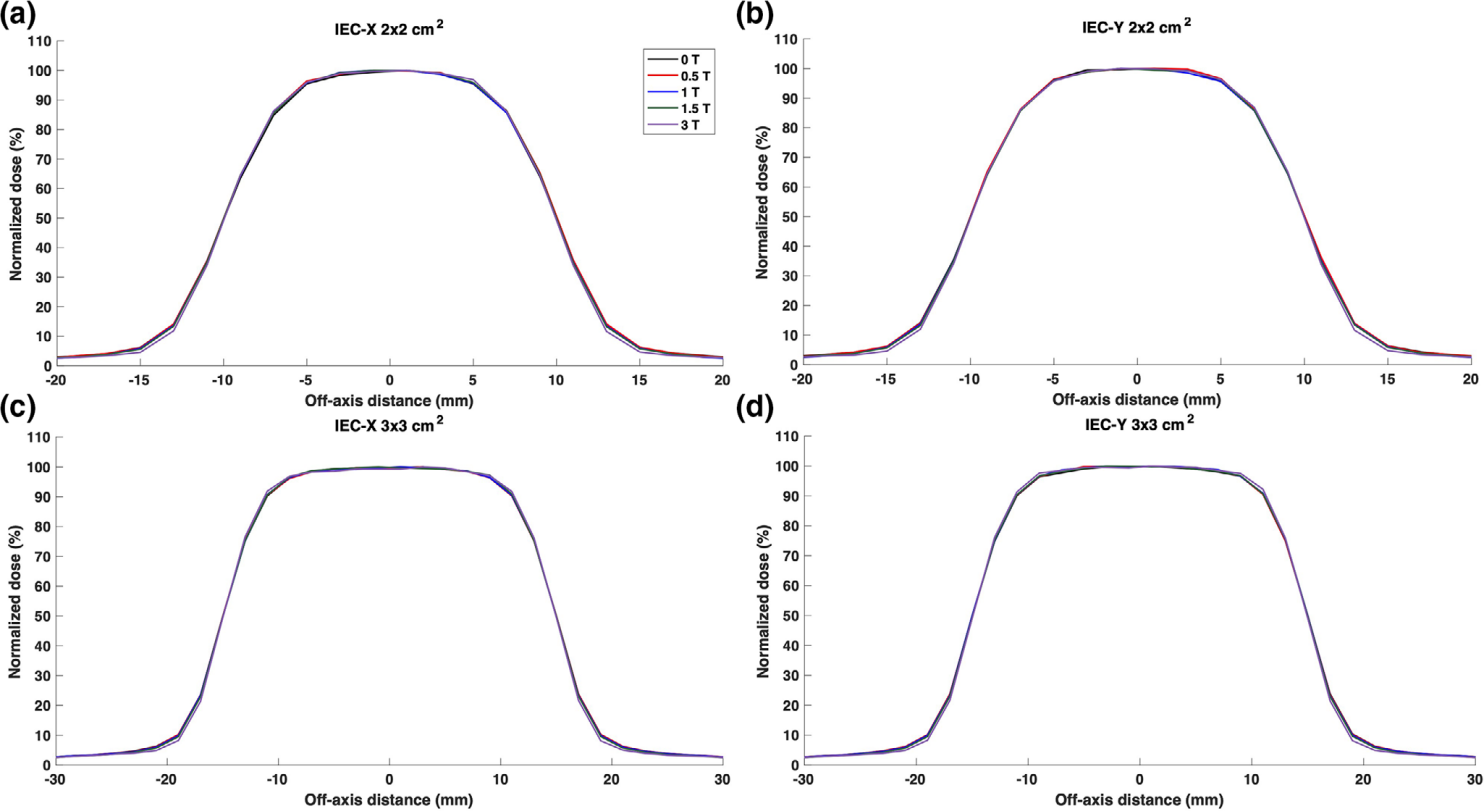
Lateral beam profiles for the 2 × 2 cm^2^ (a and b) and 3 × 3 cm^2^ (c and d) field sizes along both IEC‐X (a and c) and IEC‐Y (b and d) directions. Profiles are included for parallel magnetic field strengths ranging from 0 to 3 T. The same legend applies to all subfigures.

The $S_{clin}$ was calculated to be 0.69, 1.04, 1.99, 3.00, and 10.00 cm for the nominal 0.5 × 0.5, 1 × 1, 2 × 2, 3 × 3, and 10 × 10 cm^2^ field sizes, respectively. Figures 6 and 7 show lateral beam profiles for the small fields under the influence of parallel magnetic fields ranging from 0 to 3 T. Interestingly, the impact of the magnetic field was not statistically significant for the profile region encompassed by the 50% isodose lines across all investigated field sizes. For the 2 × 2 cm^2^ and 3 × 3 cm^2^ field sizes, no differences were found for the entire relative profiles except for a 1.5% reduction in dose in the tail region for the 3 T magnetic field. This effect was also noted for the 1 × 1 cm^2^ field size with the dose reduction in the 3 T tail region to be ∼2.5%. As displayed on Figure 6a,b for the 0.5 × 0.5 cm^2^ field size, the 3 T profile tail showed a large dose reduction of over 4% when compared to the 3 T profile. The reduction in the tail dose is indicative of the focusing effect of the magnetic field. However, this effect was found to be minimal for magnetic field strengths below 3 T.

**FIGURE 8:**
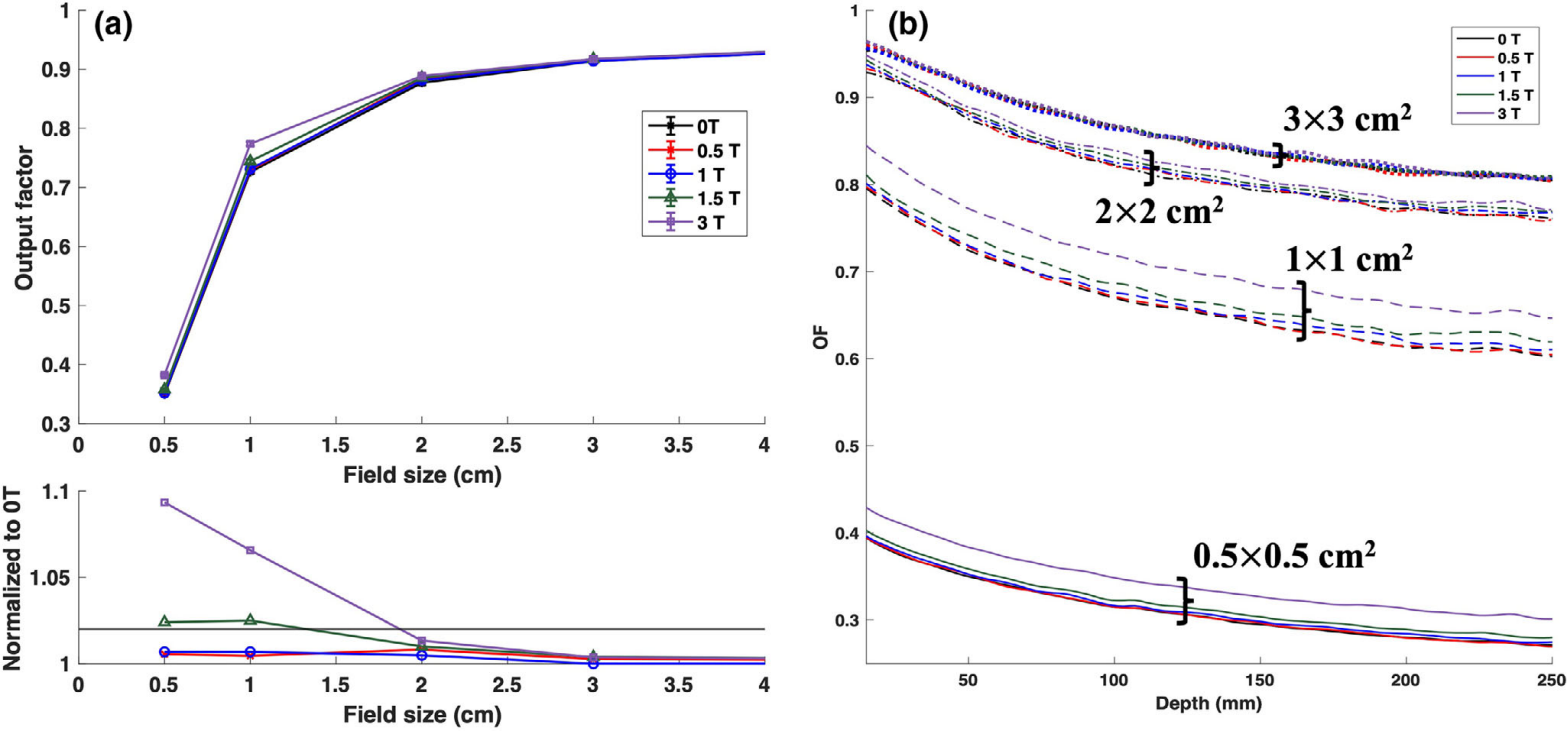
(a) Output factors (OFs) at 5 cm depth and (b) as a function of depth for parallel magnetic field strengths ranging from 0 to 3 T.

Small field output factors in water are shown in Figure 8 for both 5 cm depth and as a function of depth. The OF comparison in the build‐up region was excluded from Figure 8b for visualization purposes. For both 0.5 and 1 T, differences of <1% were found in the OFs across all small fields. These differences were negligible across all depths. Therefore, the impact of a parallel magnetic field of up to 1 T is insignificant for small field dosimetry in water except for the skin dose. For the 1.5 and 3 T magnetic field strengths, minimal differences in OFs of <1% were observed for field sizes >2 ×  2 cm^2^. However, an increase in OF was noted for the 1 × 1 cm^2^ and 0.5 × 0.5 cm^2^ field sizes. While the increase was ∼2.5% for the 1.5 T field, a dramatic increase of up to ∼9% was found for the 3 T magnetic field. This enhancement effect was noted to be consistent across all depths and is attributed to the magnetic focusing effect hypothesized for parallel MR‐linacs. As observed in Figure 6, the out of field charged particle fluence is reduced for both 1.5 and 3 T profiles for the smallest two field sizes contributing to the increase in fluence inside the field region thereby increasing the OF. Besides 3 T field, the small field conditions remain similar across various magnetic field strengths leading to differences <2.5% in a water phantom.

### 3.3 Lung phantom

**FIGURE 9:**
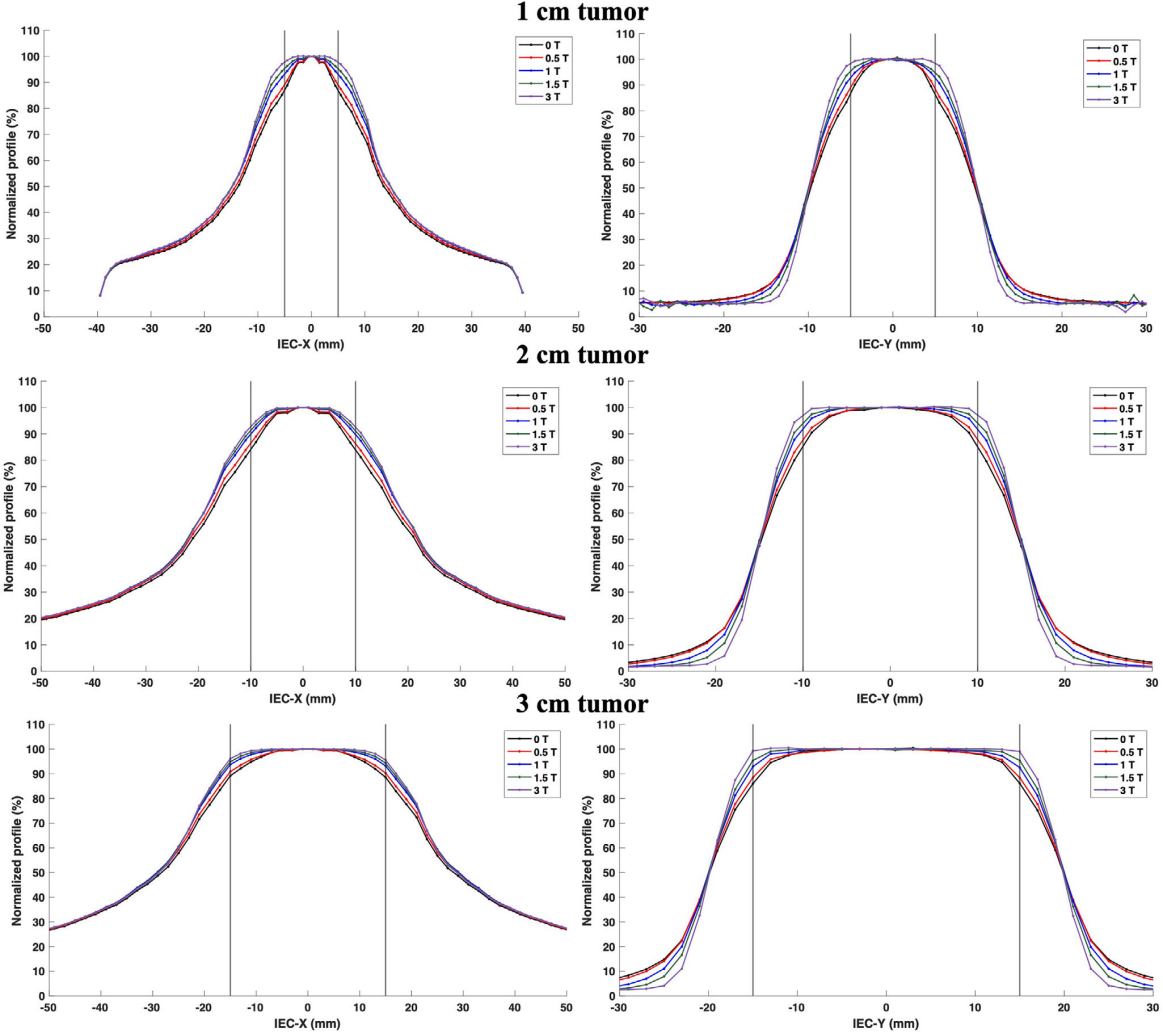
IEC‐X (left) and IEC‐Y (right) line profiles of the arc dose distribution in the lung phantom for various tumor spherical diameters for magnetic field strengths ranging from 0 to 3 T. Due to arc symmetry, IEC‐Z profile is identical to the IEC‐X profile. The vertical lines indicate the tumor boundary.

Line profiles coplanar and orthogonal to the arc rotational direction are displayed in Figure 9 for the 1, 2, and 3 cm diameter tumor spheres for varying magnetic field strengths. Due to the improved LCPE in the presence of the parallel magnetic field, an isotropic increase in the region encompassed by the 100% isodose line was observed with the increasing magnetic field strength. For the 0.5 T field strength, changes <3% were observed across all tumor sizes with the 3D dose distribution being similar to the 0 T distribution. Along the IEC‐X and IEC‐Z directions, the isodose line covering the tumor boundary increased from 87% to 98%, 84% to 93%, and 89% to 96% for the 1, 2, and 3 cm tumors, respectively. Therefore, the magnetic field impact was found to be greater for the smaller field sizes. This effect also persisted in the IEC‐Y direction with isodose line covering the tumor boundary increasing from 86% to ∼98% across the tumor sizes. A sharpening of the penumbra, defined as the distance between the 80% and 20% isodose lines, was observed in the IEC‐Y direction with the 3 T penumbra being 3 mm smaller than the 0 T penumbra. The profile tail dose was found to be decreasing with increasing magnetic field strengths along the IEC‐Y direction. The dose profiles were noted to become flatter with the increasing magnetic field strength. Such a profile shape may improve the target coverage without the presence of large hotspots inside the lesion.

**FIGURE 10:**
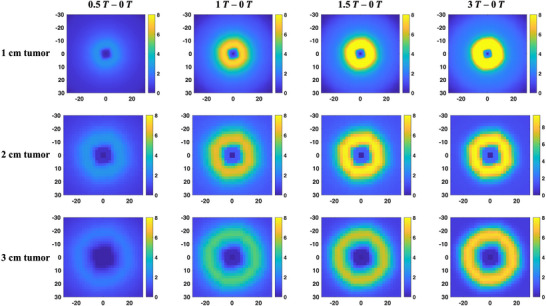
Axial 2D planar dose differences between the 0 T field and other investigated magnetic field strengths for tumor spheres with diameters of 1, 2, and 3 cm.

Planar dose differences for axial slices between the different magnetic field strengths are shown in Figure 10. The electron focusing effect due to the parallel magnetic field was found to manifest itself as a halo effect in the 2D dose distribution. As expected, dose differences increase with increasing field strength. As the tumor diameter increases, the dose differences decrease with the maximum dose differences of 13%, 9%, and 7% for the 1, 2, and 3 cm tumor diameters, respectively. The location of the peak of the annulus was observed to be consistent across all investigated magnetic field strengths and was noted to be at a distance halfway between the tumor boundary and the field edge. This trend was constant across all tumor diameters. The width of the annulus was proportional to the field strength across all tumor sizes. Therefore, the impact of magnetic field increases with increasing field strength and decreasing field size. The halo effect seen as a consequence of parallel magnetic field may improve planning target volume (PTV) coverage, especially since the PTV encompasses both the gross tumor volume (GTV) and a margin that includes lung tissue. The widening of the beam profiles due to the electron focusing effect may allow usage of smaller PTV margins. However, this halo effect can also be undesirable for target volumes abutting organs at risk (OAR). Incorporation of this halo effect in treatment planning is essential to ensure that the impact on OAR dose is accounted for.

## 4 DISCUSSION

To our knowledge, this is the first study to investigate the impact of varying magnetic field strengths on small field dosimetry of parallel MR‐linacs. The work of Huang et al. provides a comprehensive review on the impact of magnetic fields on dose distributions in MR‐linacs.^22^ However, their work focuses on orthogonal MR‐linacs while identifying the confinement of lateral scattering in a parallel MR‐linac setting. Our work provides the quantitative data on the extent of the confinement in both a water phantom and a lung phantom. Besides the 3.0 T field strength, the parallel magnetic fields were found to have a minimal impact on water phantom dosimetry to within 2%. The lung phantom study demonstrated improved uniformity of beam profiles with increasing magnetic field strength with a slight sharpening in penumbra along the direction orthogonal to the arc rotational axis. The 3.0 T field is currently not utilized by any commercial parallel MR‐linacs but can be potentially employed in the future without any dosimetric disadvantages, as demonstrated in this study. The parallel magnetic field only focuses the generated charged particles while out scattering of photons still significantly contributes to the lateral inequilbrium. The negligible change in the PDD beyond the buildup region due to the parallel magnetic field has previously been reported by both Yip et al. and Oborn et al. for larger field sizes and magnetic field strengths ranging from 0.5 to 3.0 T.^18^, ^23^ The work of Tai et al. also shows a convergence toward agreement between 0 T and 1.0 T PDD beyond the build‐up region for field sizes down to 2 × 2 cm^2^.^24^ This agreement was confirmed by our work and extended to smaller field sizes and higher magnetic field strengths. Begg et al. investigated the impact of a 1.5 T parallel magnetic field on OFs for field sizes greater than 5 × 5 cm^2^ and found minimal impact of the magnetic field on the measured OFs.^25^ Our work extended these data to small fields and reported the impact of magnetic field strengths of up to 1.5 T on OFs to be within <3%, whereas, much larger differences were reported for the 3.0 T field. A major limitation of this study includes the usage of a surrogate MC linac head model that may not be representative of commercially‐available MR‐linacs especially the Australian MR‐linac. However, our work demonstrated excellent dosimetric agreement with the Aurora‐RT system and expect the reported OF data to be close to the Australian MR‐linac system as well since both systems use a 6 MV FFF beam.^4^ The use of MC methods for small field dosimetry of the Unity MR‐linac has been previously performed by Yano et al. with good agreement with measured data.^26^ Therefore, the reported results in this study are expected to be a good representative of the experimental data.

The penumbra sharpening effect in lung tissue due to the electron focusing effect by a parallel magnetic field was previously reported by Kirkby et al. using a pencil photon beam.^10^ Similar to this work, their work reported negligible changes for in‐water beam profiles for magnetic field strengths up to 1.5 T. For a five‐field conformal lung case with parallel magnetic fields up to 3.0 T, hot spots of up to 20% around the periphery of the lung tumor were shown by Kirkby et al. when compared to 0 T dose distribution.^10^ Our study corroborates the presence of the halo hot spot effect in a lung phantom albeit with magnitudes <10%. Additionally, our study further extends the work of Kirkby et al. by showcasing the trends in the hot spot magnitude as a function of varying magnetic field strengths and tumor diameter. However, the lung phantom used in this study along with the beam arrangements are not fully representative of actual patient anatomy where differing hot spot magnitudes may be seen. The impact of tissue‐air or tissue‐bone interfaces on dose distributions is also unexplored in this work. This is a major limitation of our work and warrants further investigation. Recently, the work of Steciw et al. also reported dose perturbations at the tissue‐lung interfaces for a 0.5 T parallel magnetic field for square field sizes down to 5 × 5 cm^2^.^27^ Although a direct comparison between their study and this work is not possible due to differing phantoms and beam properties, there is agreement on the presence of the electron focusing effect in lung tissue that must be accounted for during dose calculation and treatment planning. Our work reported an increase in beam flatness as a function of magnetic field strength for the lung phantom study. Although one may hypothesize that the improved flatness can enhance the dose homogeneity inside the target for lung cases, a previous study by Pokhrel et al. concluded that the 6 MV flattened beam had similar heterogeneity index (HI) to the 6 MV FFF beam for SBRT lung cases.^28^ Therefore, accounting for the electron focusing effect in the planning optimizer may negate the differences between the 0 T and 3 T dose distributions.

## 5 CONCLUSIONS

This work investigated the impact of varying parallel magnetic fields on small field dosimetry using MC methods. The changes in the PDD, profiles, and OFs were found to be minimal, to within 2%, for field strengths up to 1.5 T. A sharp increase in the OF for the smallest fields was reported for the 3.0 T field strength. Due to the electron focusing effect being dominant in low density media, a halo effect was observed around tumor of various diameters in a lung phantom. Although the hotspot around the tumor was below 3% for the 0.5 T field strength, a consistent increase in hot spot magnitude was reported with increasing field strength by up to 8% for the 3.0 T field strength. These perturbations should be accounted for in both treatment planning and dose calculation algorithms when low density media is present.

## CONFLICT OF INTEREST STATEMENT

Ahtesham Ullah Khan has nothing to disclose.

## Data Availability

Research data are stored in an institutional repository and will be shared upon request to the corresponding author.
